# Twinning: Coronary Artery Disease in Monozygotic Twins

**DOI:** 10.7759/cureus.16139

**Published:** 2021-07-03

**Authors:** Megan C Smith, John R Baker, Evan Gleaves, Aniruddha Singh, Mohammed Kazimuddin

**Affiliations:** 1 Cardiology, University of Kentucky, Bowling Green, USA; 2 Cardiology, University of Kentucky School of Medicine, Bowling Green, USA; 3 Internal Medicine, University of Kentucky, Bowling Green, USA

**Keywords:** multivessel coronary artery disease (mvcad), genetics of coronary artery disease, monozygous twins, coronary artery intervention, coronary artery angiography

## Abstract

We present the case of a patient whose monozygotic twin brother suffered a fatal myocardial infarction at the age of 40. The patient presented with similar symptoms as his brother. Given the family history, ischemic evaluation was undertaken and revealed similar coronary anatomy and severe coronary artery disease (CAD). We review the current literature regarding genetic and environmental factors regarding coronary anatomy, locations of atherosclerotic lesions, and screening in twins.

## Introduction

Cardiovascular disease continues to be the leading cause of mortality in the United States. By 2030, 40.5% of the United States population is estimated to have coronary artery disease (CAD), with a projected direct medical cost of $818 billion [1]. Clinical practices are continuing to evolve toward understanding genetic influence on disease and the application of that data on diagnosing and treating illnesses, and preferably predicting and interceding before symptoms begin or pathology occurs. The development of CAD involves a multifaceted interaction between environmental and genetic factors, with premature CAD involving a powerful, yet not completely understood, genetic element [2]. In this report, we present a case of monozygotic twins with similar coronary anatomy and atherosclerotic lesions.

## Case presentation

A 42-year-old Caucasian man (twin A) presented to our clinic complaining of lower extremity edema and dyspnea beginning several weeks prior. His past medical history included chronic pancreatitis and insufficiency, diabetes mellitus type one, and former alcohol and cocaine abuse with last use 10 years prior. He also suffered from uncontrolled hypertension despite compliance with his medication. His medications included amlodipine 10 mg daily, lisinopril 40 mg daily, hydralazine 25 mg twice daily, and hydrochlorothiazide 25 mg daily. His family history was significant for a monozygotic twin brother (twin B) who suffered a fatal myocardial infarction (MI) at the age of 40; there was no evidence of premature CAD in any other close relative. Twin B had similar symptoms prior to MI that prompted twin A to present. The decision was made to proceed with ischemic evaluation for twin A given the family history and presenting symptoms.

**Figure 1:**
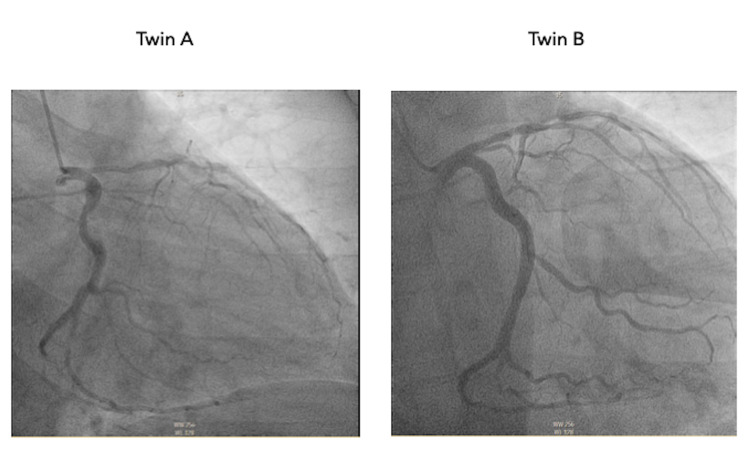
Coronary angiogram in right anterior oblique projection with caudal angulation providing visualization of the left main artery, left circumflex artery, and left anterior descending artery.

**Figure 2:**
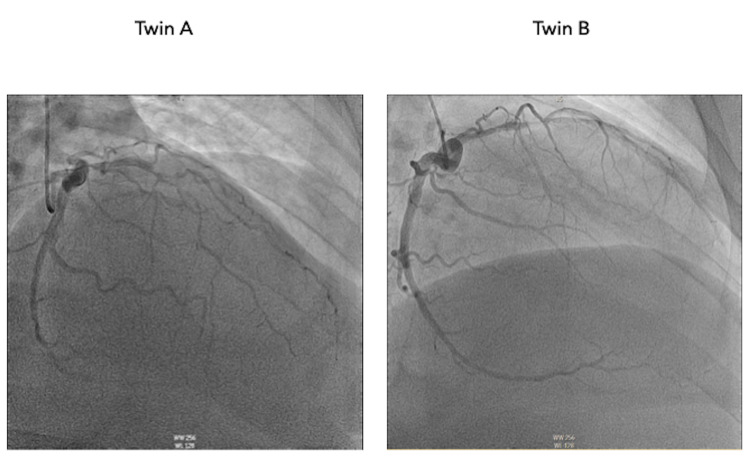
Coronary angiogram in right anterior oblique projection with cranial angulation providing visualization of the left circumflex artery and left anterior descending artery.

**Figure 3:**
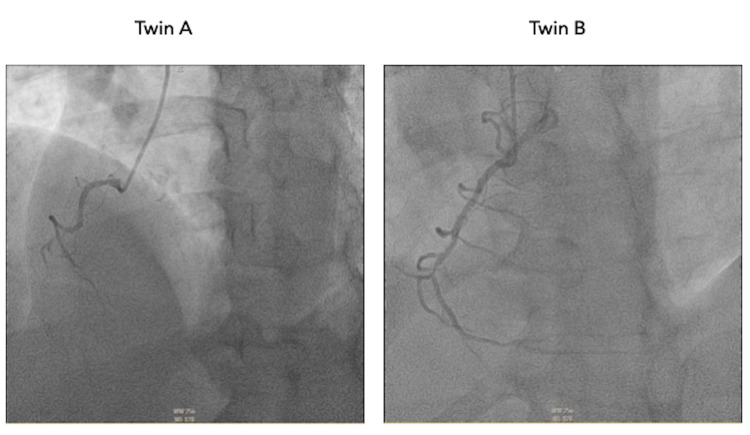
Coronary angiogram in left anterior oblique projection providing visualization of the right coronary artery.

2-D echocardiogram revealed normal ejection fraction, normal wall motion, moderate left ventricular hypertrophy, grade one diastolic dysfunction, and mild mitral regurgitation. Regadenoson stress testing revealed a large area of moderate ischemia involving the entire inferior wall and inferoseptal wall with associated moderate hypokinesis of the inferior and inferoseptal walls. This was followed by diagnostic coronary angiography which revealed a left dominant circulation and severe triple vessel CAD. There was 50% stenosis of the proximal segment and 80% focal stenosis of the distal segment of the dominant left circumflex artery (LCX), 90% mid-segment stenosis of the posterior descending artery (PDA), and 100% mid-segment occlusion of the right coronary artery (RCA). Finally, the procedure revealed ostial and proximal 40% stenosis and 90% mid-segment stenosis of the left anterior descending artery (LAD). Twin B’s coronary angiography (performed two years prior to twin A’s presentation) revealed 99% stenosis in the left PDA of the dominant LCX, 90% mid-segment stenosis of the RCA, and a prior stent occluded with thrombus in the LAD (Figures 1-3).

Twin A was referred for cardiothoracic surgery evaluation. He underwent coronary artery bypass grafting with grafts including left internal mammary artery to LAD and independent reversed saphenous vein grafts from aorta to diagonal branch and obtuse marginal branches. Eight weeks into recovery, his presenting complaints of dyspnea and lower extremity edema had completely resolved.

## Discussion

The genetic and environmental contributions to the development of CAD is not fully detailed, but investigations are currently underway. There are at least 36 loci that have been identified as susceptible to contributing to CAD due to single nucleotide polymorphisms [3]. The genetic heritability of the locations of atherosclerotic lesions in CAD is not fully known, but the preponderance of growing reports of CAD in identical twins as in our case suggests that proximal stenoses have a higher degree of heritability when compared to distal lesions [4-13], with ostial and left main CAD seemingly having the greatest genetic contribution to their frequency in monozygous twin populations [13]. Both of our patients had left-dominant coronary systems, which is present is roughly 9.1% of the population and not known to be concordant in twins [13,14]. It is possible that a different etiology for the development of proximal versus distal coronary artery atherosclerosis produces the varying heritability [15]. The high degree of similarity of lesions in our case supports the conclusions regarding the extent of heritability of CAD lesions and their locations. However, it has been proposed that an assortment of elements such as hemodynamic factors, particularly changes of arterial shear stress, are responsible for this phenomenon [16].

Despite the uncertainty regarding the heritability of atherosclerotic lesion location, the risk of CAD in a monozygotic twin whose twin has CAD, despite their personal or modifiable risk factors, is well-established. Results from a widely cited Swedish study demonstrate that if a male twin dies of a myocardial infarction, his living twin has a 50% chance of dying from CAD by the time he is 55 years of age, which is a twentyfold increase when compared to the general population [17,18]. The genetic contribution to CAD is more pronounced the younger a twin is who suffers a fatal MI, indicating an increased risk of death due to MI for the living twin. The older a twin is who suffers a fatal MI, the less likely their living twin will also die from CAD, suggesting the environmental component is a stronger contributor in those instances. Female twin data follows similar trends [18]. Regardless of young age and absence of symptoms, the likelihood of uncovering occult CAD is as high as 50% in asymptomatic twins of symptomatic patients [5], therefore serious consideration should be given towards screening asymptomatic twins of symptomatic patients [5,13,18-20] as is supported by our case.

## Conclusions

Case reports such as ours provide evidence for the genetic contribution in the development of CAD. While lesion locations are not fully dependent on genetics, certain locations do have a notable degree of heritability. The similar location of stenosis, age at first cardiac event, and risk factor profiles show concordance in this pair of identical twins. Our report highlights the importance of closely monitoring the family members of patients with CAD, particularly if they are monozygotic twins.
